# Pattern and predictors of cervical epithelial cell abnormalities among unscreened and under-screened women in Lagos, Nigeria: a cross-sectional study

**DOI:** 10.3332/ecancer.2023.1504

**Published:** 2023-02-06

**Authors:** Adeyemi Adebola Okunowo, Aloy Okechukwu Ugwu, Azeezat Oluwafunmilayo Ajose, Jubril Oladapo Kuku, Bolanle Olajumoke Okunowo, Nneoma Kwemtochukwu Ani-Ugwu, Benedetto Oluwaseun Osunwusi, Muisi Alli Adenekan, Adaiah Priscillia Soibi-Harry, Sunusi Rimi Garba

**Affiliations:** 1 Department of Obstetrics & Gynaecology, College of Medicine, University of Lagos (CMUL), Idi-Araba 102215, Lagos, Nigeria; 2 Department of Obstetrics & Gynaecology, Lagos University Teaching Hospital (LUTH), Idi-Araba 102215, Lagos, Nigeria; 3 Department of Internal Medicine, Lagos State University Teaching Hospital (LASUTH), Ikeja 101233, Lagos, Nigeria; 4 Department of Internal Medicine, Lagos University Teaching Hospital (LUTH), Idi-Araba 102215, Lagos, Nigeria; 5 Lagos Island Maternity Hospital, 10 Campbell street, Lagos Island, 102273, Lagos, Nigeria.; a https://orcid.org/0000-0002-8375-4443

**Keywords:** cervical cancer, cervical epithelial cell abnormality, cervical screening, predictors, unscreened women, under-screened women, Nigeria

## Abstract

Women who had never undergone cervical screening (CS) or who have infrequent CS are at increased risk of having cervical epithelial cell abnormalities (CECA) that may lead to cervical cancer (CCa). Our study determined the pattern and factors that predict the occurrence of CECA among unscreened and under-screened women in Lagos, Nigeria. This was an analytical cross-sectional study among 256 consenting sexually active women between 21 and 65 years who attended a community CS programme in Surulere, Lagos, Nigeria, in June 2019. Information on socio-demographic, reproductive, sexual, behavioural and clinical characteristics were collected and a Pap smear test was done. Women with abnormal cervical cytology were followed up and given appropriate treatment. Data analysis was done using Statistical Package for Social Sciences version 23. Descriptive statistics were computed using frequencies and association was tested using odd ratio. The participants’ mean age was 42.7 ± 10.3 years, majority were married (79.9%) and were human immune deficiency syndrome (HIV) negative (63.1%). The prevalence of CECA was 9.8%. Atypical squamous cell of undetermined significance and atypical squamous cell cannot exclude high-grade squamous intraepithelial lesion were the most common CECA with prevalence rates of 7.4% and 2.0%, respectively. Having a partner with multiple sexual partners (adjusted odd ratio (AOR) = 19.23), being HIV positive (AOR = 25.61), giving birth for the first time before the age of 26 years (AOR = 5.55) and presence of a combination of either abnormal vaginal discharge, contact bleeding or an unhealthy cervix on clinical examination (AOR = 13.65) independently predicted the occurrence of CECA. There is a need to prioritise CS for women with these risk factors to prevent CCa and reduce the burden of the disease in our environment.

## Introduction

Cervical cancer (CCa) has remained a major disease of public health importance in many low and middle-income countries (LMIC) especially in Sub-Saharan African countries even though it is preventable [1, 2]. It is the most common female genital tract malignancy in the region and most women present with a late-stage disease with attendant high morbidity and mortality [1]. This is due to several factors, among which are the lack of organised public health implementation of evidence-based CCa prevention strategies, poor awareness of these strategies, and consequently its non-utilisation by women [2–6]. World Health Organization (WHO) recommends prevention of CCa by primary human papillomavirus (HPV) vaccination of girls and young women preferably before sexual initiation and by regular cervical screening (CS) with HPV testing as the primary screening modality or with cytology in the absence of established HPV screening programme [7] as seen in many LMIC like Nigeria.

Before the discovery of HPV-based preventive strategies, cervical cytology (CC) played a vital role in the prevention of CCa by detecting precancerous cervical lesions (PCL) that may lead to invasive CCa. Organised screening with CC was responsible for the significant reduction in the incidence of CCa in many high-income countries [2, 4, 6]. This has made it one of the most effective screening modalities of all time [8, 9]. Despite some of its limitations in contemporary practice, it is still an essential tool for screening and early detection of PCL [7, 8].

CC primarily detects epithelial cell abnormality that leads to CCa. These cervical epithelial cell abnormalities (CECA) reflect a range of intraepithelial and epithelial lesions that represent the different grades of cervical intraepithelial neoplasm and invasive CCa, respectively [9]. The Bethesda System (TBS) was developed in 1988 for reporting CC and it has since been reviewed in 1991, 2001 and 2014 [8]. TBS reports on the presence of epithelial cell abnormality which comprises either squamous cell abnormalities or glandular cell abnormalities. The squamous epithelial abnormalities include atypical squamous cell of undetermined significance (ASCUS), atypical squamous cell cannot exclude high-grade squamous intraepithelial lesion (ASC-H), low-grade squamous intraepithelial lesion (LSIL), high-grade squamous intraepithelial lesion (HSIL) and squamous cell carcinoma (SCC). On the other hand, the glandular epithelial abnormalities include atypical endocervical, endometrial and glandular cells that favour neoplastic disease or not otherwise specified; endocervical adenocarcinoma *in situ* and adenocarcinoma [8]. Approximately 75% of CCa are of squamous cell origin [10], hence it is not surprising that squamous epithelial abnormality accounts for the majority of CECA.

The spectrum of CECA develops over a long period following high-risk HPV (hrHPV) infection. It progresses from the initial less significant low-grade squamous abnormalities such as ASCUS and LSIL which are usually self-limiting to high-grade abnormalities like HSIL which have a higher propensity to progress into CCa if not treated [9]. This underscores the need for effective CS to detect and treat CECA at the earliest stage. Women who had never undergone CS or who have infrequent CS are at increased risk of having undetected PCL that may lead to CCa. These groups of women are commonly seen in many LMICs and they contribute enormously to the persistent huge burden of CCa disease in the region [1].

In the presence of limited resources and the absence of organised CS programmes in many LMICs, it is imperative to channel some of the very limited resources in this environment towards targeted screening of high-risk women such as the unscreened or under-screened population who are at increased risk of having PCL and CCa, while efforts are geared towards making CS widely available to all women. To achieve this, it is important to identify and characterise this high-risk population and to understand the factors associated with the development of CECA in them. This will provide essential evidence-based information that would assist in the strategic screening of these women. To this end, our study aimed to determine the prevalence and pattern of CECA among the unscreened and under-screened women in Lagos, Nigeria. It also determined the socio-demographic, reproductive, sexual, behavioural and clinical factors that predicted the development of CECA in them.

## Materials and methods

### Study design and setting

The study was a cross-sectional analytical study conducted among women living in Surulere Local Government Area (LGA) of Lagos state, in June 2019. Lagos state is a metropolitan area located in the South-Western part of Nigeria and Surulere is one of its 20 LGAs. It is predominantly an urban area located in the mainland region of Lagos state. It consists of residential and commercial areas with an estimated population of 575,133 and a population density of 25,191/km^2^. It has 12 wards with a total population of 150,183 women between the ages of 21 and 65 years [11, 12]. The exact population of women within this age group living in the Shitta ward within the LGA was not available but it was estimated to be less than 10,000 by the information unit of the LGA office.

### Study population and community mobilisation

The study population was women living within the communities in the Shitta district of Surulere LGA. The CS health awareness programme was conducted within the community using a centrally located facility suggested by the community leaders that is easily accessible, appropriate for mass screening and that guarantees privacy.

Prior to the commencement of the CS programme, the research team visited the key stakeholders in the communities which included the community leaders, local residential associations, leaders of different women-led community groups, women societies, religious and traditional leaders to explain the purpose of the CS exercise, the activities involved, their anticipated engagement and the benefits to the community. Permission was obtained to carry out the screening exercise and community women were sensitised and invited to attend the CS health awareness programme through these stakeholders.

## Recruitment of study participants

Women who attended the health awareness programme were informed about the purpose of the study and were progressively recruited into the study as they came for the programme by convenient sampling method. Sexually active women between 21 and 65 years without prior history of PCL or CCa who gave informed consent were enrolled in the study. Women younger than 21 years old, who had never been sexually active, who were menstruating or unwilling to participate in the study were excluded from the study. Sample size was calculated using appropriate formula (*n* = *Z*^2^ *p* (1 − *p*)/*d*^2^) [13] with an absolute error margin of 5% (*d* = 0.05), type 1 error of 5% (*Z* = 1.96), and local prevalence of CECA (*p*) of 13.9% [14]. The calculated minimum sample size was 184 and after adjusting for a non-response rate of 15%, the final sample size was 212.

### Instrument of survey and data collection

A structured questionnaire was used to obtain relevant information on socio-demographic, reproductive, sexual, behavioural and clinical characteristics from the study participants. Information obtained on socio-demographic characteristics were age, tribe, religion, education and marital status. The sexual and reproductive information obtained were age at coitarche, age at first childbirth, number of deliveries, number of lifetime sexual partners, history of partner having other sexual partners and history of sexually transmitted infection (STI). Similarly, information on age at marriage, type of marriage, history of smoking, use of oral contraceptive pills (OCP) and history of previous CS were elicited under behavioural characteristics. The clinical information obtained from the participants included their human immune deficiency virus (HIV) status, history or presence of abnormal vaginal discharge, contact bleeding and unhealthy cervix on vaginal examination. For the purpose of the study, all women who had never undergone CS before were regarded as ‘unscreened’ while women who had only been screened once in their lifetime were regarded as ‘under-screened’.

The questionnaire was pretested prior to the commencement of the study for the appropriateness of its content and clarity of its instructions among a convenient sample of 30 women who attended the gynaecological outpatient clinic at Lagos University Teaching Hospital (LUTH). The outcome of the pilot study was used to revise the final study questionnaire as appropriate. Questionnaires were administered by trained interviewers and data were collected from all eligible participants after informed consent was obtained. CS with conventional Pap smear was done for all eligible participants after clinical inspection of the cervix for abnormalities.

### Cervical screening procedure and sample collection

Conventional Pap smear was used for the CS procedure. CS was conducted in a secured room in the presence of a female chaperone after obtaining informed consent. Participants were positioned in a dorsal position on the examination couch, covered and appropriately sized Cusco’s speculum inserted to expose the vaginal and cervix. Excessive mucus and discharge were wiped with a cotton swab soaked with normal saline after observation of the findings. A cervical brush was inserted into the external cervical os and rotated 360° to obtain cells from the transformation zone. These were transferred to the glass slide by rolling the bristles across the glass slide. The slides were then sprayed with spray fixative, allowed to air dry, kept in a jar and transported to the laboratory.

### Clinical management

Women with identifiable gynaecological problems were given appropriate medical treatment or referral for further evaluation and treatment. Results of the Pap smear were communicated to the women and women with abnormal cytology had further evaluation and treatment.

### Laboratory procedure

The Pap staining was done using the progressive pap staining method. This is a polychromatic staining technique involving the use of haematoxylin solution, orange green 6 solution and eosin azure solutions which is a mixture of eosin Y, light green SF and Bismarck brown dyes. Following wet fixation with 95% alcohol for a minimum of 15 minutes, the slides were hydrated in 80%, 70% and 50% ethanol, respectively, and rinsed gently with distilled water. Harris haematoxylin was applied for 2–3 minutes and rinsed with water. They were dipped in 0.5% hydrochloric acid thrice and then placed under running tap water for 5 minutes. The slides were dehydrated by inserting them in distilled water, 50%, 70%, and 80% ethanol, respectively. Counterstain with orange-green 6 solution for 2 minutes and thereafter rinsed three times in 95% alcohol by dipping it six times in the alcohol solution per each rinse. The slides are again counterstained with eosin alcohol-50 solution for 2 minutes and rinsed thrice again with 95% alcohol. They are dehydrated with absolute alcohol for 1 minute and finally cleared using xylene solution [15].

For quality assurance, the slides were prepared by trained cytotechnicians following the standard recommended protocol and reviewed independently by two cytopathologists and by a third cytopathologist if the two reports were different. Pap smears were reported using the current Bethesda System (2014) for reporting CC.

### Data analysis

The data was de-identified, cleaned and validated. The data was analysed using Statistical Package for Social Sciences version 23.0, IBM Corp., Armonk, NY, USA. Descriptive statistics were computed for all data and presented as frequencies and percentages in tables or charts. Quantitative data was checked for normality using Shapiro–Wilk’s test. Normally distributed continuous variables were presented as mean ± standard deviation (SD) while skewed continuous variables were expressed as the median and interquartile range (IQR). Qualitative and quantitative data were grouped into categories for ease of analysis. Bivariate analysis was done using Pearson’s chi-square test (or Fischer’s exact test when the expected cell value was less than 5) and Student’s *t*-test (or Mann–Whitney *U* test) to compare categorical and continuous variables, respectively. Univariate regression analysis was done by computing a crude odd ratio (COR) to assess the degree of association between CECA and explanatory variables. Multivariable regression modelling was done by including variables with *p*-value < 0.20 on univariate analysis. Binary logistic regression using the stepwise backward elimination technique was used to build the final model. Statistical significance was set at a two-tailed *p*-value < 0.05 and a 95% confidence interval.

## Results

Among the 256 eligible women that participated in the study, 244 (95.3%) women had complete data and were included in the analysis. Out of these, only 4.5% (11/244) of the participants had ever undergone CS while 95.5% (233/244) were unscreened. All of the screened participants had only been screened once in their lifetime.

### Characteristics of study participants

Table 1 shows the characteristics of the participants. Majority (42.2% (103/244)) of the participants were between the ages of 31 and 40 years with a mean age of the 42.7 ± 10.3 years (range: 21–65 years). Most of the participants had tertiary education (84.4% (206/244)), were married (79.9% (195/244)), Christians (94.7% (231/244)) and approximately half were of Yoruba ethnicity (48.0% (117/244)).

The median parity of the women was 3 (2–5). The mean age at coitarche was 22.7 ± 2.9 years and the mean age at first childbirth was 28.1 ± 3.2 years. Majority (59.8% (146/244)) of the participants had a total of between 2 and 3 lifetime sexual partners with median sexual partners of 2 (2–3) and only 15.2% (37/244) had ever had STI.

Table 1 further shows the behavioural and clinical characteristics of the women in the study. Most (86.6% (194/224)) of the participants that were married were in a monogamous relationship and got married at a mean age of 27.3 ± 3.1 years. Only 12.7% (31/244) of the study participants smoked cigarettes while 38.9% (95/244) and 2.9% (7/244) used OCP and were positive for HIV, respectively. Only 21.3% (52/244) of the participants had vaginal discharge, contact bleeding or an unhealthy cervix on clinical examination.

### Prevalence and pattern of CECA

**Figure 1.**
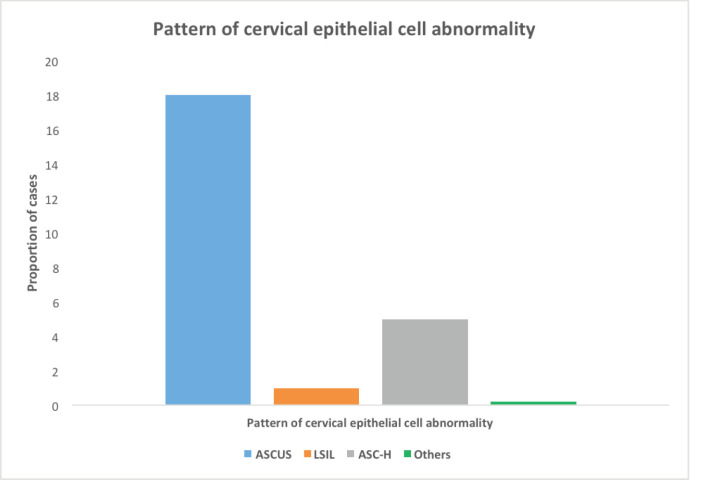
Pattern of cervical epithelial cell abnormality. *Others include HSIL, glandular abnormalities and cervical cancer.

The prevalence of CECA among the participants was 9.8% (24/244). ASCUS accounted for 75% (18/24) of CECA, with a prevalence rate of 7.4% (18/244) followed by ASC-H which accounted for 20.8% (5/24) of CECA with a prevalence rate of 2.0% (5/244) and LSIL accounting for 4.2% (1/24) of all the abnormalities with a prevalence rate of 0.4% (1/244). None of the participants had HSIL, glandular abnormality or CCa (Figure 1).

### Factors associated with CECA

None of the socio-demographic factors were significantly associated with CECA even though the majority of the participants with CECA were Christians (95.8% (23/24)), married (70.8% (17/24)) with tertiary education (79.2% (19/24)) and within the ages 31–40 years (58.3% (14/24)) (*p* > 0.05, respectively). Having more than one-lifetime sexual partner and partners who have other sexual partners were significantly associated with having CECA (*p* = 0.049 and *p* < 0.001, respectively). Parity, age at coitarche and age at first childbirth were not significantly associated with having CECA (*p* > 0.05, respectively). Among the behavioural, and clinical factors, only previous use of OCP, HIV status, presence of abnormal vaginal discharge, contact bleeding, unhealthy cervix or a combination of any of these clinical findings were found to be significantly associated with having CECA (*p* = 0.040, 0.001, <0.001, <0.001, 0.001 and <0.001, respectively) (Table 2).

### Univariate and multivariate predictors of CECA

Table 3 shows the univariate and multivariate predictors of CECA among the participants. Giving birth to first child before 26 years (COR = 3.91, CI = 1.61–9.52, *p* = 0.004), having a partner who has other sexual partners (COR = 10.50, CI = 3.04–36.26, *p* < 0.001), previous use of OCP (COR = 2.40, CI = 1.02–5.66, *p* = 0.040) and being HIV positive (COR = 14.47, CI = 3.02–69.22, *p* = 0.002) were significantly associated with increased odds of developing CECA on univariate regression analysis. Similarly, the presence of vaginal discharge (COR = 5.88, CI = 2.43–14.22, *p* < 0.001), contact bleeding (COR = 15.90, CI = 5.36–47.14, *p* < 0.001), unhealthy cervix (COR = 10.14, CI = 3.08–33.40, *p* = 0.001) or a combination of either of these conditions (COR = 13.29, CI = 2.12–34.46, *p* < 0.001) were significantly associated with increased odds of having CECA. However, women who were married or had been married before had significantly decreased odds of developing CECA compared to their counterparts who had never been married (COR = 0.28, CI = 0.09–0.85, *p* = 0.034).

After modelling for all the significant variables and variables with *p*-value < 0.20, only having a partner who had other sexual partners (adjusted odd ratio (AOR) = 19.23, CI = 3.36–110.10, *p* = 0.001), being HIV positive (AOR = 25.61, CI = 2.60–252.0, *p* = 0.005), giving birth to first child before 26 years (AOR = 5.55, CI = 1.63–18.94, *p* = 0.006) and a combination of either abnormal vaginal discharge, contact bleeding or unhealthy cervix (AOR = 13.65, CI = 4.26–43.70, *p* < 0.001) were significant independent predictors of the occurrence of CECA among the study participants.

## Discussion

CECA are a spectrum of early to late cervical intraepithelial lesions that may result in CCa if not detected and treated early. Early identification of CECA through regular CS remains a major hallmark of CCa prevention. As a result, under-screened or unscreened women are at high risk of having undetected CECA and consequently CCa. Our study investigated the prevalence of CECA; the socio-demographic, reproductive, sexual, behavioural and clinical factors that influenced and predicted its occurrence among unscreened and under-screened women in Lagos, Nigeria. The prevalence of CECA among the studied population was 9.8% with ASCUS and ASC-H being the most common with a prevalence rate of 7.4% and 2.0%, respectively. Having a partner who has other sexual partners, being HIV positive, having childbirth before the age of 26 years and the presence of a combination of either abnormal vaginal discharge, contact bleeding or an unhealthy cervix on clinical examination significantly predicted the occurrence of CECA.

Almost all of the participants (95.5%) had never had CS in their lifetime while the few who had been screened were under-screened. This finding highlights the huge burden of poor CS practices among women in our region, which has been a great challenge to CCa prevention in LMIC over the years [16]. Several studies within Lagos [2, 17–19], other parts of Nigeria [20–22] and LMIC [16, 23–25] are consistent with the low CS rate reported in our study, and in contrast to that reported in developed countries [26, 27]. This is probably due to the lack of organised screening programmes in many LMIC compared to what is obtained in developed countries resulting in opportunistic screening of few women [2, 3]. In addition, poor awareness about the benefits of CS, methods of CS, inadequate information on CS, fear of being diagnosed with CCa, limited access to CS services, poverty and infrequent recommendation of CS by clinicians [2–4, 20] are the possible reasons for the observed low rate of CS in our environment. Consequently, there is a need to urgently scale up the practice and uptake of CS in the country and other LMICs to reduce the burden of CCa and possibly eliminate it, in line with WHO’s vision [28]. This may be possible by encouraging motivators and drivers of CS uptake such as increasing public awareness about CCa and the benefits of CS, making CS more accessible and affordable or free for women and regular counselling of women who come to the health facility by healthcare practitioners on the need to undergo CS [2, 20].

The prevalence of CECA among the participants was 9.8%. This shows some similarities and discrepancies with the rates reported in different parts of Nigeria and other LMICs. In Nigeria, the prevalence rate of CECA varies from 5%–11.3% in the northern region [29, 30], 11.2%–16.5% in the eastern region [31, 32] to 13.9%–34.6% in the western region [14, 33]. Similar variation was observed in many LMICs with reported rates of 0.2%–12% in India [34, 35], 2.5%–5.7% in Saudi Arabia [36], 3.7% in Ghana [37], 3.7% in Oman [38], 3.8% in Jordan [39], 4.4% in Kuwait [40], 5.1% in Turkey [41], 8.7% in Tanzania [42] and 14.1% in Ethiopia [43]. Several factors have been shown to account for this variation. These include the rate of STI, HIV/acquired immune deficiency syndrome (AIDS), HPV infections and screening practices among women. Women with a high rate of these infections have an increased risk of CECA compared to others [35, 43, 44]. Similarly, women who had never been screened or who screen infrequently are at higher risk of having CECA. However, the prevalence of CECA was relatively low among the study participants despite their poor screening practice. Similar low prevalence of 0.2% and 3.7% was also reported by Ghosh *et al* [34] and Donkoh *et al* [37] in India and Ghana, respectively. This is probably due to the low prevalence of high-risk conditions such as HIV/AIDS and STI among the participants which are known to influence the occurrence of PCL.

ASCUS was the most common CECA, accounting for three-quarters of all the CECA, with a prevalence rate of 7.4%. This is similar to the rate reported in other studies [38–40, 43]. It is generally considered the most common CECA with prevalence ranging between 1.6% and 9.0% [45]. ASC-H, another form of atypical squamous cell abnormality was the second most common CECA detected in our study. ASCUS and ASC-H are atypical squamous cells that closely resemble LSIL and HSIL, respectively, but do not strictly meet its diagnostic criteria. They are usually a reflection of inflammatory, reactive or repair changes, but may also be due to HPV-related precancerous lesions [45]. Some studies have however reported LSIL [16, 29, 30, 32, 37, 42] and HSIL [31, 35] as the most commonly detected CECA during cytology. This is contrary to our findings where LSIL and HSIL were the least prevalent. Generally, low-grade epithelial cell abnormalities are more common than high-grade abnormalities.

Several factors were found to be associated with having CECA in our study and this included marital status, age at first childbirth, having partners who have other sexual partners and use of OCP. Others include HIV status, findings of vaginal discharge, contact bleeding, an unhealthy-looking cervix or a combination of any of the three features. However, age at first childbirth, having partners who have other sexual partners, being HIV positive and a combination of any of the symptoms of abnormal vaginal discharge, contact bleeding or unhealthy cervix were the only independent factors that significantly predicted CECA.

The odds of developing CECA were 26 times higher among HIV-positive women compared to those who are not. Other studies have also reported a higher prevalence among HIV-positive women compared to their counterparts [35, 43]. This is not surprising as HIV/AIDS has been a major risk factor for the development of PCL and CCa due to its association with HPV and immunosuppressive state [35, 37, 42, 44]. The level of CD4 count and stage of HIV/AIDS disease has also been shown to influence the development of PCL and CCa [42, 43]. It is generally recommended that HIV/AIDS women commence CS at an earlier age and undergo a more frequent and longer period of screening.

Sexual activity is a critical factor in the acquisition of HPV which causes PCL and CCa. Age at coitarche [29, 43], having more than one sexual partner [29, 31, 32, 43, 44] and having partners with other sexual partners [46] have been reported to influence the risk of PCL and CCa. However, in our study, having partners with multiple sexual partners was the only sexual factor that significantly predicted the risk of CECA, while similar to findings in other studies [31, 32, 44] age at coitarche and multiple sexual partners did not. This may be due to the relatively older age at sexual initiation observed among our study participants. Women who had partners with multiple sexual partners were 19 times more likely to develop CECA compared to women whose partners do not have other sexual partners. This highlights the important role sexual partners play in modifying the risk of their partners’ developing CECA and possibly CCa. Partners that are either promiscuous or have multiple sexual partners may aid transmission of hrHPV and STIs to their partners and thus increase their risk of developing PCL and CCa.

Age at first childbirth significantly predicted the occurrence of CECA in our study. This is consistent with its association with CCa [47]. The likelihood of developing CECA was approximately six times higher among women who had their first childbirth below 26 years compared to those above that age. Conversely, parity was not associated with having CECA in our study. Women with high parity were not at higher risk of CECA compared to those with lower parity. This is congruent to findings in some studies [32, 38, 44] but contrary to findings in others [30, 31, 36, 43].

PCL and CECA are usually asymptomatic and detected accidentally during screening; however, some clinical conditions may be associated with them. We found that women who have a combination of any of the clinical findings of abnormal vaginal discharge, contact bleeding and unhealthy cervix were at an increased risk of developing CECA compared to women who do not have any symptoms. This is congruent to findings in other studies where these clinical conditions have been reported among women with abnormal smears or CECA [16, 31, 36, 43]. This underscores the need for health education about the risk posed by the presence of these conditions, especially in unscreened women, and to ensure that women who have these symptoms undergo regular CS. Our study did not find any significant association between CECA and socio-demographic factors contrary to other studies where age [14, 30, 32, 42, 44], occupation [32, 43] and marital status [32] were significantly associated with its occurrence.

The Society of Gynaecology and Obstetrics of Nigeria recommends that women, in general, should start CS at 25 years, while women at high risk for developing CCa as determined by the physician should commence CS before 25 years [48]. There is a need for policymakers and physicians to recognise the significant risk of CECA associated with women giving birth before 26 years, having partners who have multiple sexual partners, being HIV positive and having cervicovaginal symptoms and signs. These high-risk women will benefit from starting CS before 25 years. Similarly, women with cervicovaginal symptoms and signs of vaginal discharge, contact bleeding or unhealthy cervix should often be considered for CS as part of their normal clinical evaluation, especially in the absence of a recent CS.

### Strengths and limitations

A major strength of our study is its ability to assess multiple factors including clinical factors that affect the risk of developing CECA among a predominantly unscreened population of women who are at high risk of developing CCa. The method used for data collection may have some inherent weaknesses such as recall bias and response bias. It is assumed that the respondents provided truthful and accurate information when responding to the questions provided by the interviewers. All the interviewers were trained on the data collection process. The interviews were conducted in private and comfortable rooms and the questions were asked in a simple and non-biased manner, and time was given to allow for adequate recall in order to reduce recall or response bias. Another limitation in our study is its relatively small sample size which is probably responsible for the large confident interval observed in some of its explanatory variables like HIV status during univariate and multivariate analyses. This implies an imprecise estimate of the effect of the variable on the outcome measured and may not be representative of the general population. Overall, the study provided useful insights into the predictive factors for CECA among high-risk women in Lagos, Nigeria. However, larger population-sized studies are needed to further investigate these findings.

## Conclusion

The prevalence of CECA among predominantly unscreened Nigerian women was 9.8%. ASCUS and ASC-H epithelial abnormalities were the most common CECA. Age at first childbirth below 26 years, having partners who have other sexual partners, being HIV positive and presence of a combination of vaginal discharge, contact bleeding or unhealthy cervix were independent predictors of CECA. There is a need to prioritise CS for these women to prevent the risk of PCL and CCa.

## List of abbreviations

AIDS, Acquired immune deficiency syndrome; ASC-H, Atypical squamous cell cannot exclude high-grade squamous intraepithelial lesion; ASCUS, Atypical squamous cell of undetermined significance; AOR, Adjusted odd ratio; CC, Cervical cytology; CCa, Cervical cancer; CECA, Cervical epithelial cell abnormality; CS, Cervical screening; COR, Crude odd ratio; HIV, Human immune deficiency syndrome; HPV, Human papillomavirus; hrHPV, High-risk HPV; HSIL, High-grade squamous intraepithelial lesion; LGA, Local Government Area; LMIC, Low and middle-income countries; LSIL, Low-grade squamous intraepithelial lesion; OCP, Oral contraceptive pills; PCL, Precancerous cervical lesions; SCC, Squamous cell carcinoma; STI, Sexually transmitted infection; TBS, The Bethesda System; WHO, World Health Organization.

## Conflicts of interest

The authors declare no conflicts of interest.

## Funding

This research received no funding.

## Ethical approval

Ethical approval (ADM/DCST/HREC/APP/2870) was obtained from the Human Research and Ethical committee of LUTH before conducting the study. Informed consent was obtained from all participants before their enrolment in the study. The study was carried out in accordance with the Declaration of Helsinki (1964).

## Figures and Tables

**Table 1.** Characteristics of participants.

Variables	Frequency (%) *n* = 244
Socio-demographic characteristics	
Age	
21–30	20 (8.2)
31–40	103 (42.2)
41–50	66 (27.0)
51–60	34 (13.9)
>60	21 (8.7)
Mean age ± SD	42.7 ± 10.3
Education	
None	6 (2.5)
Primary	1 (0.4)
Secondary	31 (12.7)
Tertiary	206 (84.4)
Marital status	
Single	20 (8.2)
Married	195 (79.9)
Separated	12 (4.9)
Divorced	1 (0.4)
Widowed	16 (6.6)
Ethnicity	
Hausa	8 (3.3)
Ibo	115 (47.1)
Yoruba	117 (48.0)
Others	4 (1.6)
Religion	
Christianity	231 (94.7)
Islam	13 (5.3)
Reproductive and sexual characteristics	Frequency (%) *n* = 244
Parity	
0	16 (6.6)
1–3	124 (50.8)
>3	104 (42.6)
Median parity (IQR)	3 (2–5)
Age at coitarche	
<21	64 (26.2)
21–25	140 (57.4)
26–30	37 (15.2)
>30	3 (1.2)
Mean age ± SD	22.7 ± 2.9
Age at first childbirth	*n* = 227
≤25	44 (19.4)
26–30	146 (64.3)
31–35	30 (13.2)
>35	7 (3.1)
Mean age ± SD	28.1 ± 3.2
Number of lifetime sexual partners	
1	43 (17.6)
2–3	146 (59.8)
>3	55 (22.6)
Median (IQR)	2 (2–3)
Partners with other sexual partners	
Yes	109 (44.7)
No	135 (55.3)
History of STI	
Yes	37 (15.2)
No	207 (84.8)
Behavioural and clinical characteristics	Frequency (%) *n* = 224
Age at marriage	
<21	1 (0.5)
21–25	56 (25.0)
26–30	138 (61.6)
31–35	25 (11.1)
>35	4 (1.8)
Mean age ± SD	27.3 ± 3.1
Type of marriage	
Monogamous	194 (86.6)
Polygamous	30 (13.4)
Smoking	
Yes	31 (12.7)
No	213 (87.3)
Use of OCP	
Yes	95 (38.9)
No	149 (61.1)
HIV status	
Positive	7 (2.9)
Negative	154 (63.1)
Not sure	83 (34.0)
Presence of vaginal discharge	
Yes	44 (18.0)
No	200 (82.0)
Presence of contact bleeding	
Yes	17 (7.0)
No	227 (93.0)
Presence of unhealthy cervix	
Yes	13 (5.3)
No	231 (94.7)
Presence of either vaginal discharge, contact bleeding or unhealthy cervix	
Yes	52 (21.3)
No	192 (78.7)

**Table 2.** Association between socio-demographic, sexual, reproductive, behavioural and clinical characteristics and cervical epithelial cell abnormality. Bold values indicate 95% significant level.

Socio-demographic characteristics	Epithelial cell abnormality		*p* value
	Yes	No	
Age (*n* = 244)	*n* = 24 (%)	*n* = 220 (%)	
21–30	1 (4.2)	19 (8.6)	0.547
31–40	14 (58.3)	89 (40.5)	
41–50	5 (20.8)	61(27.7)	
51–60	3 (12.5)	31 (14.1)	
>60	1 (4.2)	20 (9.1)	
Education (*n* = 244)	*n* = 24 (%)	*n* = 220 (%)	
None	2 (8.3)	4 (1.8)	0.276
Primary	0 (0.0)	1 (0.5)	
Secondary	3 (12.5)	28 (12.7)	
Tertiary	19 (79.2)	187 (85.0)	
Marital status (*n* = 244)	*n* = 24 (%)	*n* = 220 (%)	
Single	5 (20.8)	15 (6.8)	0.147
Married	17 (70.8)	178 (80.9)	
Separated	2 (8.3)	10 (4.5)	
Divorced	0 (0.0)	1 (0.5)	
Widowed	0 (0.0)	16 (7.3)	
Ethnicity (*n* = 244)	*n* = 24 (%)	*n* = 220 (%)	
Hausa	1 (4.2)	7 (3.2)	0.956
Ibo	12 (50.0)	103 (46.8)	
Yoruba	11 (45.8)	106 (48.2)	
Others	0 (0.0)	4 (1.8)	
Religion (*n* = 244)	*n* = 24 (%)	*n* = 220 (%)	
Christianity	23 (95.8)	208 (94.5)	1.000
Islam	1 (4.2)	12 (5.5)	
**Sexual and reproductive characteristics**	**Epithelial cell abnormality**		***p* value**
	**Yes**	**No**	
Parity (*n* = 244)	*n* = 24 (%)	*n* = 220 (%)	
0	1 (4.2)	15 (6.8)	0.950
1–3	13 (54.2)	111 (50.5)	
>3	10 (41.6)	94 (42.7)	
Age at coitarche (*n* = 244)	*n* = 24 (%)	*n* = 220 (%)	
<21	3 (12.5)	61 (27.7)	0.335
21–25	16 (66.7)	124 (56.4)	
26–30	5 (20.8)	32 (14.5)	
>30	0 (0.0)	3 (1.4)	
Age at first childbirth (*n* = 227)	*n* = 23 (%)	*n* = 204 (%)	
≤25	10 (43.5)	34 (16.7)	0.103
26–30	10 (43.5)	136 (66.7)	
31–35	2 (8.7)	28 (13.7)	
>35	1 (4.3)	6 (2.9)	
Number of lifetime sexual partners (*n* = 244)	*n* = 24 (%)	*n* = 220 (%)	
1	2 (8.3)	41 (18.6)	**0.049**
2–3	12 (50.0)	134 (60.9)	
>3	10 (41.7)	45 (20.5)	
Partners with other sexual partners (*n* = 244)	*n* = 24 (%)	*n* = 220 (%)	
Yes	21 (87.5)	88 (40.0)	**<0.001**
No	3 (12.5)	132 (60.0)	
History of STI (*n* = 244)	*n* = 24 (%)	*n* = 220 (%)	
Yes	4 (16.7)	33 (15.0)	1.000
No	20 (83.3)	187 (85.0)	
**Behavioural and clinical characteristics**	**Epithelial cell abnormality**		***p* value**
	**Yes**	**No**	
Age at marriage (*n* = 224)	*n* = 19 (%)	*n* = 205 (%)	
<21	0 (0.0)	1 (0.5)	0.518
21–25	3 (5.8)	53 (25.9)	
26–30	15 (78.9)	123 (60.0)	
31–35	1 (5.3)	24 (11.6)	
>35	0 (0.0)	4 (2.0)	
Type of marriage (*n* = 224)	*n* = 19 (%)	*n* = 205 (%)	
Monogamous	16 (84.2)	178 (86.8)	0.744
Polygamous	3 (15.8)	27 (13.2)	
Smoking (*n* = 244)	*n* = 24 (%)	*n* = 220 (%)	
Yes	5 (20.8)	26 (11.8)	0.329
No	19 (79.2)	194 (88.2)	
Use of OCP (*n* = 244)	*n* = 24 (%)	*n* = 220 (%)	
Yes	14 (58.3)	81 (36.8)	**0.040**
No	10 (41.7)	139 (63.2)	
HIV status (*n* = 244)	*n* = 24 (%)	*n* = 220 (%)	
Positive	4 (16.7)	3 (1.4)	**0.001**
Negative	14 (58.3)	140 (63.6)	
Not sure	6 (25.0)	77 (35.0)	
Presence of vaginal discharge (*n* = 244)	*n* = 24 (%)	*n* = 220 (%)	
Yes	12 (50.0)	32 (14.5)	**<0.001**
No	12 (50.0)	188 (85.5)	
Presence of contact bleeding (*n* = 244)	*n* = 24 (%)	*n* = 220 (%)	
Yes	9 (37.5)	8 (3.6)	**<0.001**
No	15 (62.5)	212 (96.4)	
Presence of unhealthy cervix (*n* = 244)	*n* = 24 (%)	*n* = 220 (%)	
Yes	6 (25.0)	7 (3.2)	**0.001**
No	18 (75.0)	213 (96.8)	
Combination of vaginal discharge, contact bleeding or unhealthy cervix (*n* = 244)	*n* = 24 (%)	*n* = 220 (%)	
Yes	18 (75.0)	34 (15.5)	**<0.001**
No	6 (25.0)	186 (84.5)	

**Table 3.** Predictors of cervical epithelial abnormality.

Variables	Univariate predictors			Multivariate predictors		
	COR	CI	*p*-value	AOR	95% CI	*p*-value
Age (years)						
>40	0.58	0.24–1.38	0.212	-	-	-
≤40	1					
Education						
Secondary and below	1.49	0.52–4.27	0.551	-	-	-
Tertiary	1					
Marital status						
Ever married	0.28	0.09–0.85	**0.034**	-	-	-
Never married	1					
Ethnicity						
Yoruba	0.91	0.39–2.12	0.827	-	-	-
Others	1					
Religion						
Others	0.75	0.09–6.06	1.000	-	-	-
Christianity	1					
Parity						
>3	0.96	0.41–2.25	0.921	-	-	-
≤3	1					
Age at coitarche (years)						
<20	0.50	0.11–2.21	0.397	-	-	-
≥20	1					
Age at first childbirth (years)						
≤25	3.91	1.61–9.52	**0.004**	5.55	1.63–18.94	**0.006**
>25	1					
More than one lifetime sexual partners						
Yes	2.52	0.57–11.14	0.269	-	-	-
No	1					
Partners with other sexual partners						
Yes	10.50	3.04–36.26	**<0.001**	19.23	3.36–110.10	**0.001**
No	1					
History of STI						
Yes	1.13	0.36–3.53	1.000	-	-	-
No	1					
Age at marriage (years)						
≤25	0.63	0.21–1.93	0.463	-	-	-
>25	1					
Type of marriage						
Polygamous	1.49	0.47–4.71	0.511	-	-	-
Others	1					
Smoking						
Yes	1.96	0.68–5.71	0.329	-	-	-
No	1					
Use of OCP						
Yes	2.40	1.02–5.66	**0.040**	-	-	-
No	1					
HIV status						
Positive	14.47	3.02–69.22	**0.002**	25.61	2.60–252.00	**0.005**
Others	1					
Presence of vaginal discharge alone						
Yes	5.88	2.43–14.22	**<0.001**	-	-	-
No	1					
Presence of contact bleeding alone						
Yes	15.90	5.36–47.14	**<0.001**	-	-	-
No	1					
Presence of suspicious or unhealthy cervix alone						
Yes	10.14	3.08–33.40	**0.001**	-	-	-
No	1					
Presence of combination of vaginal discharge, contact bleeding or unhealthy cervix						
Yes	13.29	2.12–34.46	**<0.001**	13.65	4.26–43.70	**<0.001**
No	1					

COR, Crude odd ratio; AOR, Adjusted odd ratio; CI, Confidence interval
